# Phase II study to evaluate the efficacy of Trastuzumab in combination with Capecitabine and Oxaliplatin in first-line treatment of HER2-positive advanced gastric cancer: HERXO trial

**DOI:** 10.1007/s00280-019-03820-7

**Published:** 2019-03-29

**Authors:** Fernando Rivera, C. Romero, P. Jimenez-Fonseca, M. Izquierdo-Manuel, A. Salud, E. Martínez, M. Jorge, V. Arrazubi, J. C. Méndez, P. García-Alfonso, M. Reboredo, J. Barriuso, N. Muñoz-Unceta, R. Jimeno, C. López

**Affiliations:** 1 Medical Oncology Department, Hospital Universitario Marqués de Valdecilla, Avda Valdecilla SN, 39008 Santander, Spain; 2 Medical Oncology Department, POVISA, Vigo, Spain; 3 Medical Oncology Department, Hospital Universitario Central de Asturias, Oviedo, Spain; 4 Medical Oncology Department, Hospital Arnau de Vilanova, Lleida, Spain; 5 Medical Oncology Department, Hospital Xeral Cies, Vigo, Spain; 6 Medical Oncology Department, Complejo Hospitalario de Navarra, Pamplona, Spain; 7 Medical Oncology Department, Centro Oncológico de Galicia, Coruña, Spain; 8 Medical Oncology Department, Hospital Gregorio Marañón, Madrid, Spain; 9 Medical Oncology Department, Complejo Hospitalario de A Coruña, Coruña, Spain; 10 Medical Oncology Department, Hospital La Paz, Madrid, Spain

**Keywords:** Advanced gastric cancer, HER2 positive, Phase II study, Oxaliplatin, Capecitabine, Trastuzumab

## Abstract

**Purpose:**

The phase III ToGA trial established cisplatin, fluoropyrimidine and trastuzumab as the standard treatment in HER2-positive advanced gastric cancer (AGC). However, as demonstrated in HER2-negative AGC, oxaliplatin-based regimens could improve tolerance remaining effective. The aim of this trial was to explore the potential activity and safety of capecitabine, oxaliplatin (XELOX) and trastuzumab in patients with HER-2 positive advanced gastric cancer.

**Methods:**

We conducted a multicentre, prospective, non-randomised, non-controlled, open-label and national (Spanish) phase II study. Patients with HER2-positive advanced gastric or gastro-oesophageal junction (EGJ) cancer received XELOX and trastuzumab as first-line treatment. Primary endpoint was objective tumour response rate (ORR).

**Results:**

45 patients from ten hospitals in Spain were included from September 2011 to December 2013. Median age was 65 years, 82.2% were male, 69% had gastric cancer and 31% had EGJ tumours. At a median follow-up of 13.7 months (7.1–20.9), the estimated median progression-free survival and overall survival were 7.1 (95% CI 5.5–8.7) and 13.8 months (95% CI 10.1–17.4), respectively, with 8.9%, 37.8% and 31.1% of patients achieving complete response, partial response and stable disease. Regarding safety, 44.4% of the patients had grade 3 or greater adverse events, being the most frequent diarrhoea (26.6%), fatigue (15.5%), nausea (20%) and vomiting (13.3%). Only two patients (4.4%) developed asymptomatic grade 2 left ventricle ejection fraction reduction.

**Conclusions:**

XELOX-trastuzumab is a promising and effective therapy as first-line treatment for patients with HER2-positive AGC, with comparable results to the ones obtained with other “platinum-based” regimens. This scheme is feasible and tolerable with a low incidence of cardiac toxicity.

## Introduction

Gastric cancer (GC) is the second most commonly diagnosed cancer in the world. There are important geographical differences in terms of incidence and mortality. In Europe, 140,000 new cases were diagnosed in 2012. During the same year, 107,000 patients died, being the fourth leading cause of cancer deaths [1].

One of the reasons for such a high mortality rate in GC is the difficulties for an early diagnosis, and its high relapse rate even in resectable tumours. Despite significant advances in the treatment of advanced disease (metastatic, unresectable locally advanced or relapsed disease), its prognosis remains very poor. Therefore, new therapeutic options need to be investigated.

The ToGA trial, a phase III, open-label, randomised controlled study showed that patients with human epidermal growth factor receptor 2 (HER2)-positive oesophagogastric junction (EGJ) or advanced gastric cancer (AGC) receiving trastuzumab plus capecitabine/5FU and cisplatin had better outcomes compared to patients receiving chemotherapy alone. Median overall survival (OS) was 13.8 months (95% confidence interval [CI] 12–16) for patients assigned to trastuzumab plus chemotherapy arm compared with 11.1 months (95% CI 10–13) in the chemotherapy group (hazard ratio [HR] 0.74; 95% CI 0.60–0.91; *p* = 0.0046). In a preplanned overall survival analysis performed in patients with HER2 immunohistochemistry + + positive (IHC 2+) and fluorescence in situ hybridisation (FISH) positive or IHC 3 + tumours, there was an increase of 5 months in median OS in favour of the experimental arm (16.8 vs 11.8 months, HR: 0.65) [2]. Based on these findings, the combination of trastuzumab with cisplatin and fluoropyrimidine could be considered as a standard treatment in patients with advanced gastric or EGJ HER2-positive adenocarcinomas [3].

Two phase III trials, REAL-2 [4] and ML17032 [5], have suggested that capecitabine has equivalent efficacy and better tolerability than 5FU and can replace it in the treatment of AGC.

REAL2 trial also showed that oxaliplatin has similar efficacy and less toxicity than cisplatin, being possible to use it in this setting [4]. Another phase III trial from Al-Batran et al. achieved the same conclusion [6].

The combination of capecitabine and oxaliplatin (XELOX) was explored in the adjuvant setting in a phase III study (CLASSIC) [7] and nowadays can be considered as a standard regimen for this indication. Moreover, although this combination has not yet been studied in a phase III trial of patients with AGC patients, several phase II trials suggest that XELOX has an interesting activity and good tolerance as first-line therapy [8–10].

Preclinical data suggest that trastuzumab promotes thymidine phosphorylase (TP) expression. This enzyme plays a main role in the activation process of capecitabine. Therefore, the combination of trastuzumab and capecitabine might be interesting in the treatment of HER2-positive GC [11]. A French group has explored the combination of oxaliplatin–5FU with trastuzumab in this population, showing promising activity [12]. Two phase II trials, both performed specifically in Asiatic population, have reported a favourable toxic profile and high efficacy of XELOX–trastuzumab [13, 14]. However, data concerning the use of this chemotherapy scheme as first-line regimen for metastatic or unresectable gastric and EGJ HER2-positive cancer are still limited, especially in western countries.

In light of all these results, we have conducted a multicentre, prospective, non-randomised, non-controlled, open-label and national (Spanish), phase II study to assess the efficacy and safety of trastuzumab in combination with XELOX as first-line treatment in patients with HER2-positive advanced gastric or EGJ cancer.

## Materials and methods

### Patient eligibility

Patients diagnosed with HER2-positive unresectable locally advanced, recurrent and/or metastatic GC or EGJ histologically confirmed adenocarcinoma, were eligible. We considered as HER2-positive tumours, primary or metastatic samples with HER2 overexpression defined by immunohistochemistry +++ (IHC3+) or ++ (ICH2+) confirmed by FISH/SISH (FISH/SISH+).

Included patients had measurable disease according to the Response Evaluation Criteria In Solid Tumors (RECIST) version 1.1, age was ≥ 18 years, Eastern Cooperative Oncology Group (ECOG) score was 0–2, life expectancy was more than 3 months, and they had adequate renal, liver and bone marrow function.

Enrolled patients should be able to take tablets orally and should not have undergone surgery within 4 weeks previous to study inclusion. Patients who had received previous oxaliplatin or previous chemotherapy for GC were excluded (except adjuvant/neoadjuvant chemotherapy completed at least 6 months before enrolment).

Active and clinically significant cardiovascular disease, defined as uncontrolled hypertension, unstable angina, congestive heart failure grade II or higher of the New York Heart Association (NYHA), severe cardiac arrhythmias that require medication or peripheral vascular disease stage II or higher, was considered as exclusion criteria. A normal left ventricle ejection fraction, (LVEF) > 50% measured by echocardiography or mitigated acquisition (MUGA) scan, was mandatory.

Patients with previous or recurrent history of other malignancies within the last 5 years prior to treatment start were excluded (except curatively treated basal cell carcinoma of the skin or in situ carcinoma of the cervix).

The study was approved by the Ethics Review Board at each institution and by the Spanish Agency of Medicines and Medical Devices (AEMPS). All procedures performed in the trial were in accordance with the 1964 Helsinki declaration and its later amendments. Written informed consent was obtained from all individual participants included before any specific study procedure was undertaken.

### Treatment

Trastuzumab (Herceptin^®^, Roche Pharmaceuticals) was administered intravenously (i.v.) with a loading dose of 8 mg/kg on day 1 followed by 6 mg/kg every 3 weeks. Capecitabine (Xeloda ®, Roche Pharmaceuticals) was administered orally (p.o.) at a dose of 1000 mg/m^2^/12 h on days 1–14 every 3 weeks during six cycles. Oxaliplatin (Eloxatin ®, Sanofi Pharmaceuticals) was administered i.v. at a dose of 130 mg/m^2^ in a 2-h infusion on day 1, every 3 weeks, during six cycles (XELOX-Trastuzumab). After six chemotherapy cycles, trastuzumab monotherapy was maintained until disease progression or intolerable toxicity. Dose adjustments were allowed at all times to manage toxicity.

### Assessments and statistical analysis

Primary endpoint was objective tumour response rate (ORR) according to RECIST version 1.1. Tumour assessments were evaluated at baseline (within 21 days prior to the administration of the first dose of study treatment) and every three cycles (9 weeks) by computed tomography (CT) or magnetic resonance imaging (MRI), until disease progression. Response to treatment was defined as complete response (CR), partial response (PR), stable disease (SD) or progressive disease (PD). ORR was defined as the sum of CR and PR.

Secondary endpoints included OS, progression-free survival (PFS) and safety. OS was defined from the first day of treatment until the date of death and PFS was estimated from the first day of treatment to the date of progressive disease (PD) or death. Survival was calculated using the Kaplan–Meier method, providing the median, 95% CI and number of both events and censored cases.

Adverse events (AE), related and unrelated to study medication (trastuzumab, capecitabine and oxaliplatin), were collected during treatment and every 28 days after the last dose of study drug. This evaluation consisted of description, maximum severity reached, resolution, and causality of AE. Particularly LVEF, determined by echocardiography or MUGA scan, was performed after the six cycles of XELOX–trastuzumab, every 4 months of trastuzumab treatment and when trastuzumab was stopped for any reason.

All analyses were performed on the intention to treat (ITT) population. Data were analysed using SPSS 18.0 version.

## Results

### Patient characteristics

From September 2011 to December 2013, a total of 45 patients (ITT population) from 10 hospitals at the north of Spain were included. Cut-off for data analysis was carried out on July 1, 2015.

Main baseline characteristics of patients included are reported in Table 1.

**Table 1:** Patient characteristics at baseline

Patient baseline characteristics	*N* = 45	%
**Median age, years (range)**	65 (44–80)	
**Sex**		
Men	37	82
Women	8	18
**Race**		
Caucasian	45	100
**ECOG score**		
0	19	42.2
1	21	46.7
2	5	11.1
**Extent of disease at study entry**		
Relapse after curative resection	7	16
Metastatic	37	82
Unresectable locally advanced	1	2
**Previous chemotherapy (neoadjuvant or adjuvant)**		
Yes	3	6.7
No	42	93.3
**Metastatic sites**		
Liver	28	62.2
Lung	11	24.4
Peritoneum	9	20
Lymph nodes	25	55.6
Other	7	15.6
**Histologic subtypes**		
Intestinal	25	55.5
Diffuse	8	17.8
Undetermined	12	26.7
**Immunohistochemistry (IHC)**		
2+ (confirmed by SISH + in all cases)	12	26.7
3+	33	73.3

### Efficacy

Tumour response analysis was performed in the ITT population: 4 patients (8.9%) achieved CR, 17 patients (37.8%) had PR and 14 patients (31.1%) showed SD according to RECIST v 1.1 criteria. ORR was 46.7% (95% CI 31.9–62.0). Two patients discontinued treatment before the administration of the third cycle so they could not be radiological evaluated. Median duration of response in 21 patients with CR or PR was 9.4 months (95% CI 5.5–13.3) and median time to achieve this response was 2.3 months (95% CI 2.0–2.5).

**Fig. 1:**
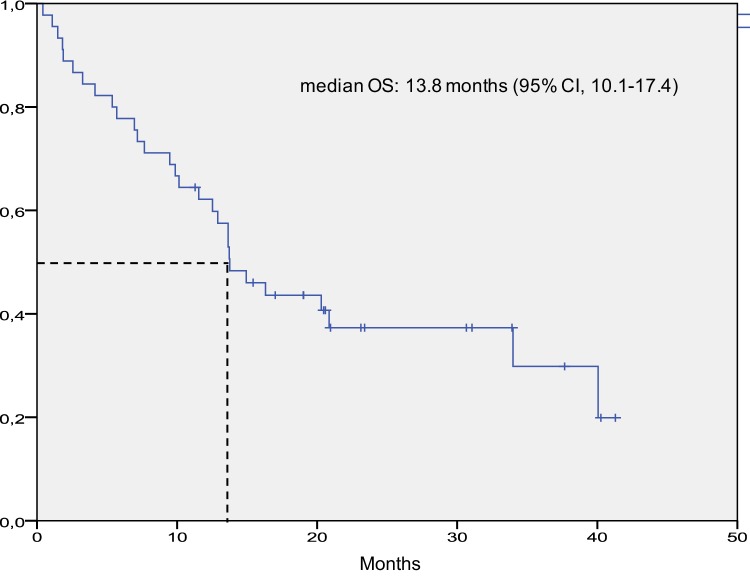
Kaplan–Meier curve for overall survival in the intention-to-treat population. *CI* denotes confidence interval

**Fig. 2:**
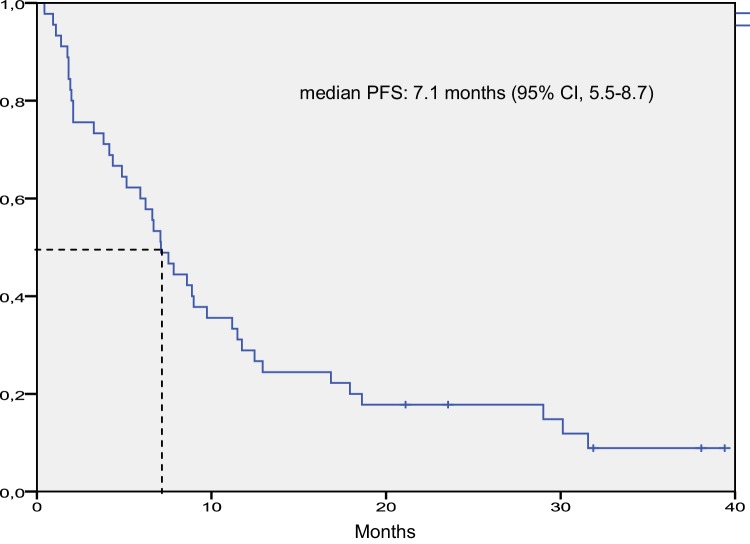
Kaplan–Meier curve for progression-free survival in the intention-to-treat population. *CI* denotes confidence interval

Median follow-up was 13.7 months (7.1–20.9 months). Median OS was 13.8 months (95% CI 10.1–17.4) and median PFS was 7.1 months (95% CI 5.5–8.7) for the entire population (Figs. 1 and 2).

### Safety

Toxicity is shown in Table 2. Overall, 42 out of 45 patients (93.3%) experienced any AEs during treatment. However, only 20 (44.4%) patients developed grade 3 or greater AEs. The most frequent grade 3/4 toxicities were diarrhoea (26.6%), fatigue (15.5%), nausea (20%), vomiting (13.3%), hyporexia (6.7%), neurotoxicity (2.2%), anaemia (2,2%) and neutropenia (2.2%). No cases of neutropenic fever were recorded.

**Table 2:** Treatment-related adverse events. LVEF: left ventricle ejection fraction. HTA: hypertension

Adverse events	Grade I		Grade II		Grade III		Grade IV	
	*n*	%	*n*	%	*n*	%	*n*	%
LVEF reduction	0	0	2	4.4	0	0	0	0
HTA	1	2.2	1	2.2	0	0	0	0
Heart failure	0	0	0	0	1	2.2	0	0
Hand–foot syndrome	5	11.1	0	0	1	2.2	0	0
Fatigue	13	28.9	13	28.9	6	13.3	1	2.2
Diarrhoea	4	8.9	8	17.8	11	24.4	1	2.2
Nausea	4	8.9	8	17.8	8	17.8	1	2.2
Vomiting	8	17.8	3	6.7	6	13.3	0	0
Mucositis	2	4.4	3	6.7	1	2.2	0	0
Hyporexia	4	8.9	1	2.2	1	2.2	0	0
Anaemia	11	24.4	5	11.1	1	2.2	0	0
Neutropenia	3	6,7	6	13.3	1	2.2	0	0
Thrombocytopenia	7	15.5	5	11.1	0	0	0	0
Neurotoxicity	22	48.8	12	26.6	1	2.2	0	0

Only two patients (4,4%) developed asymptomatic grade 2 LVEF reduction and one patient was diagnosed with clinically relevant congestive heart failure.

Oxaliplatin dose was reduced in 17 patients due to AEs: thrombocytopenia (4), weight loss (3), neutropenia (1) and non-hematologic adverse events (9) such as neurotoxicity. Capecitabine dose was adjusted in 16 patients during the study because of diarrhoea (8), thrombocytopenia (1) and others (7). Of note, trastuzumab dose was decreased in eight patients because of weight loss.

## Discussion

In this phase II trial, we found that XELOX–trastuzumab is an effective scheme with an acceptable toxicity profile as first-line treatment for advanced HER2-positive gastric or EGJ cancers.

Comparing our data with the results obtained with trastuzumab and cisplatin–5FU–capecitabine therapy at the ToGA trial, they seem very similar. Respectively, ORR is 46.7% and 47%, median PFS is 7.1 and 6.7 months and OS is 13.8 and 13.8 months. Nevertheless, we have to consider that patients in the ToGA trial were younger than our population (median age 59 vs 65 years old) and they included 51% of Asian patients.

Regarding toxicity, XELOX–trastuzumab was well tolerated and had a similar rate of haematologic AEs than in ToGA trial. However, although grade 1–2 neurotoxicity, diarrhoea and hand–foot syndrome were slightly more frequent in our experience, no clinically significant differences were observed regarding grade 3–4 toxicities. Cardiac toxicity was an infrequent event in both studies. Chemotherapy-related toxicity observed in our patients was similar to that described in other trials that have explored the XELOX combination in patients with gastric cancer in the adjuvant setting [7] or in advanced disease [8–10].

Two other phase II trials exploring the combination of XELOX–trastuzumab in advanced HER2-positive GC patients have been published. Both have been developed in Asia and have shown a promising activity and a tolerable toxicity profile with this regimen. Phase II trial from Ryu et al. [13] included 55 patients with HER2-positive AGC and reported 68% ORR and 89% Disease Control Rate (DCR). Median PFS and OS were 8.6 and 21 months, respectively. The toxicity reported in this study was moderate, similar to the one usually described with XELOX. No significant trastuzumab-related toxic effects (including cardiac toxicity) were reported. In another phase II trial from Gong et al. [14], XELOX–trastuzumab also showed similar efficacy (ORR: 66.7%; DCR: 86.3%; median PFS: 9.2 months and median OS: 19.5 months) in the 51 patients included. The toxicity was again mild and similar to that previously reported with XELOX, also without significant trastuzumab-related side effects. The efficacy observed in these two phase II trials with XELOX–trastuzumab seems to be slightly higher than that observed in our trial. One reason could be the existence of differences in the characteristics of the populations included. Both Asian trials included more patients with locally advanced disease (4% in the Ryu trial, 13.7% in the Gong trial and 2% in our trial), median age was lower (57, 57 and 65 years old, respectively), and less patients had ECOG 2 (7%, 5.9% and 11.1%, respectively). Another possible difference could be the proportion of patients HER2 positive by IHC (IHC 3+: 89%, 74.5% and 73.3%, respectively) and there are also some differences in the distribution of Lauren histological variants (intestinal: 66% vs 55%; diffuse: 19% vs 17%; mixed or unspecified: 13% vs 26% in the Gong trial and in our study, respectively). These differences are common among Asian and Western populations and could justify, at least partially, these results.

As previously mentioned, our trial mainly included patients with metastatic (82%) or relapsed disease (16%). Only 2% of patients had locally advanced disease. The ToGA trial also included mostly metastatic patients (97%) and the same happened in the trial of Ryu et al. [13] previously referred exploring XELOX–trastuzumab. The role of trastuzumab combined with chemotherapy should be properly explored in the unresectable locally advanced setting and specific clinical trials focused on this population should be performed.

In the early stage GC, XELOX–trastuzumab was explored in the Spanish phase II trial NEOXH [15] as peri-operative treatment in 36 patients with HER2-positive gastric or EGJ resectable adenocarcinoma. Toxicity was similar to that observed in our trial and efficacy was promising: 18-month PFS rate (primary endpoint) was 71%; 24-month PFS rate was 60%; 24-month OS rate was 75%; pathological complete response was 8%; and R0 resection rate was 78%. Finally, the phase II clinical trial INNOVATION is currently exploring the addition of trastuzumab (± pertuzumab) to pre-operative chemotherapy in this setting.

According to all these previous data, XELOX regimen could be an interesting partner for anti-HER-2 treatment in patients with HER2-positive GC.

## Conclusions

XELOX–trastuzumab in the first-line therapy for patients with HER2-positive AGC showed promising and effective results, comparable in terms of ORR to the ones obtained with other “platinum-based” schemes. There are some differences between Asian and Western patients that could have an impact in the OS differences observed with these combinations.

This regimen is feasible and tolerable with a low incidence of cardiac toxicity.

We need to design specific investigational protocols for Western population to explore the potential role of this combination for patients with HER2-positive AGC at earlier stages (adjuvant or neoadjuvant therapy).
