# Correction to: Phase II study to evaluate the efficacy of Trastuzumab in combination with Capecitabine and Oxaliplatin in first-line treatment of HER2-positive advanced gastric cancer: HERXO trial

**DOI:** 10.1007/s00280-019-03964-6

**Published:** 2019-10-05

**Authors:** Fernando Rivera, C. Romero, P. Jimenez-Fonseca, M. Izquierdo-Manuel, A. Salud, E. Martínez, M. Jorge, V. Arrazubi, J. C. Méndez, P. García-Alfonso, M. Reboredo, J. Barriuso, N. Muñoz-Unceta, R. Jimeno, C. López

**Affiliations:** 1 Medical Oncology Department, Hospital Universitario Marqués de Valdecilla and Instituto de Investigación Marqués de Valdecilla (IDIVAL), Avda Valdecilla SN, 39008 Santander, Spain; 2 Medical Oncology Department, POVISA, Vigo, Spain; 3 Medical Oncology Department, Hospital Universitario Central de Asturias, Oviedo, Spain; 4 Medical Oncology Department, Hospital Arnau de Vilanova, Lleida, Spain; 5 Medical Oncology Department, Hospital Xeral Cies, Vigo, Spain; 6 Medical Oncology Department, Complejo Hospitalario de Navarra, Pamplona, Spain; 7 Medical Oncology Department, Centro Oncológico de Galicia, Coruña, Spain; 8 Medical Oncology Department, Hospital Gregorio Marañón, Madrid, Spain; 9 Medical Oncology Department, Complejo Hospitalario de A Coruña, Coruña, Spain; 10 Medical Oncology Department, Hospital La Paz, Madrid, Spain

## Correction to: Cancer Chemotherapy and Pharmacology (2019) 83:1175–1181 10.1007/s00280-019-03820-7

The article “Phase II study to evaluate the efficacy of Trastuzumab in combination with Capecitabine and Oxaliplatin in first-line treatment of HER2-positive advanced gastric cancer: HERXO trial”, written by Fernando Rivera, C. Romero, P. Jimenez–Fonseca, M. Izquierdo–Manuel, A. Salud, E. Martínez, M. Jorge, V. Arrazubi, J. C. Méndez, P. Garcia–Alfonso, M. Reboredo, J. Barriuso, N. Munoz–Unceta, R. Jimeno, C. López was originally published electronically on the publisher’s internet portal (currently SpringerLink) on 29th March 2019 without open access.

With the author(s)’ decision to opt for Open Choice the copyright of the article changed on 5th April 2019 to © The Author(s) 2019 and the article is forthwith distributed under the terms of the Creative Commons Attribution 4.0 International License (http://creativecommons.org/licenses/by/4.0/), which permits use, duplication, adaptation, distribution and reproduction in any medium or format, as long as you give appropriate credit to the original author(s) and the source, provide a link to the Creative Commons license and indicate if changes were made.

The original article has been corrected.

